# Vincristine-induced acute life-threatening hyponatremia resulting in seizure and coma

**DOI:** 10.4103/0972-5229.58545

**Published:** 2009

**Authors:** Mahesh Nagappa, Ravindra R. Bhat, K. Sudeep, Sandeep Kumar Mishra, A. S. Badhe, B. Hemavathi

**Affiliations:** **From:** Department of Anesthesiology and Critical Care, JIPMER, Pondicherry, India

**Keywords:** Hyponatremia, seizures, Vincristine, Wilms tumor

## Abstract

We report a case of a four-year-old boy with stage 1 Wilms tumour, who developed Vincristine-induced acute life-threatening hyponatremia, which presented as generalized tonic clonic seizures and coma. He was intubated and mechanically ventilated. There were no localizing neurological signs. CSF study showed no cells and CSF proteins were 20 mg%. Electrocardiography, chest X-ray, echocardiography, CT scan and liver function tests were normal. Evaluation of electrolytes and arterial blood gas showed serum sodium of 113 mEq/L with mild metabolic acidosis. Serum osmolality was 260 mOsm/L (normal value 285-295 mOsm/L) and urine osmolality was 625 mOsm/L (normal range 300-900 mOsm/L), urine sodium 280 mEq/d (normal range 100-260 mEq/d), serum potassium, blood urea, blood sugars were normal. Serial blood cultures showed no bacterial growth. Patient was treated with fluid restriction, hypertonic saline (3%) and other supportive care. Patient improved clinically over three days and was extubated on the third day and shifted to the ward on the fifth day.

## Introduction

Vincristine (VCR) is a naturally occurring vinca alkaloid that blocks mitosis by arresting cells in the metaphase. VCR is delivered via intravenous infusion for use in various types of chemotherapy regimes. Its main uses are in the treatment of non-Hodgkin's lymphoma, Hodgkin's lymphoma, acute lymphoblastic leukemia (ALL) and nephroblastoma (Wilms tumour, a renal tumour common in children). The main adverse effects of VCR are peripheral neuropathy, hyponatremia, constipation and hair loss. Around 96 cases of hyponatremia and/or SIADH associated with VCR have been reported. We herein report a case of Wilms tumour, who developed VCR-induced acute life threatening hyponatremia presenting as seizures and coma.

## Case Report

A 4-year old boy was diagnosed with stage 1 Wilms tumour. Chemotherapy was initiated according to the protocol using Vincristine (1.5 mg/m^2^) on days 1 and 5 and Actinomycin D (0.5 mg/m^2^) on days 1-5 was to be repeated every 3 months till the completion of the cycles. The peripheral blood film before starting the second cycle showed a hemoglobin of 11 g%, TC 21,000/ mm^3^ and platelets 2,00,000/ mm^3^, Blood urea 20 mg%, Blood sugar 94 mg%, Sodium 138 mEq/L, Potassium 4.0 mEq/L. On day 5 after the administration of the two drugs due for the day (VCR and Actinomycin D), he was initially noted to be drowsy, then had an episode of generalized tonic clonic seizure and became comatose. There were no localizing neurological signs. He was intubated and mechanically ventilated. Electrolytes taken at this time showed a serum sodium of 113 mEq/L with mild metabolic acidosis and normal PaCo_2_. Serum osmolality was 260 mOsm/L (normal value 285-295 mOsm/L), urine osmolality was 625 mOsm/L (normal range 300-900 mOsm/L) and urine sodium 280 mEq/d (normal range 100-260 mEq/d). Serum potassium, blood urea and blood sugar were normal. Over the next three days sodium correction was done with fluid restriction supplemented with 3% hypertonic saline. Serum sodium improved over three days to 135 mEq/L. CSF study showed no cells, CSF proteins were 20 mg%. Electrocardiography, Chest X-ray, Echocardiography, CT scan and liver function tests were normal.

## Discussion

In our patient, hyponatremia appeared on the fifth day of the second cycle of chemotherapy and improved within 3 days of treatment. Since the other organ functions were normal and the cardinal feature of SIADH was present, hyponatremia was attributed to VCR.

The reported rate of SIADH associated with VCR is very low, around 1.3/100,000 treated patients. Schwartz[1] had reported the first case of SIADH. Fine *et al.*,[2] had reported the first case of SIADH with VCR therapy. The average age of the patients who present with this side effect was 35 ± 28 years, 62% were males. Hammond *et al.*,[3] had showed that the racial distribution is predominantly in Asians. According to Stuart *et al.*,[4] SIADH usually occurs between 4 and 10 days after VCR administration and improves within 1 week of starting symptomatic treatment. The severity and frequency of SIADH depends on the frequency and doses of VCR administered. When the serum sodium falls below 115, altered mental status, confusion, lethargy, psychosis, seizures, coma and occasionally death may occur. Rarely, focal neurologic signs are present. Risk factors for VCR-induced SIADH include Asian patients,[3] patients with liver disease,[5] HIV patients[6] and elderly patients.[7]

Symptomatic treatment of SIADH associated with VCR is mainly based on fluid restriction supplemented with administration of 3% hypertonic saline solution and diuresis with intravenous furosemide.
